# Review of the Structural Characteristics and Biological Activities of *Tricholoma* Secondary Metabolites (2018–2023)

**DOI:** 10.3390/molecules29194719

**Published:** 2024-10-05

**Authors:** Meili Zhao, Shiqin Yuan, Zhiming Li, Chengwei Liu, Ruiying Zhang

**Affiliations:** 1 Key Laboratory for Enzyme and Enzyme-Like Material Engineering of Heilongjiang, College of Life Science, Northeast Forestry University, Harbin 150040, China; 17349876786@163.com (M.Z.); 18845128317@163.com (S.Y.); lzm110103@163.com (Z.L.); 2 State Key Laboratory of Efficient Utilization of Arid and Semi-Arid Arable Land in Northern China, The Institute of Agricultural Resources and Regional Planning, Chinese Academy of Agricultural Sciences, Beijing 100081, China

**Keywords:** *Tricholoma*, secondary metabolites, terpenoids, anti-cancer, mushroom

## Abstract

*Tricholoma* are significant medicinal and edible mushrooms within Basidiomycota. Known for their various medicinal properties such as anti-tumor, immune regulation, and antioxidant effects, they are regarded worldwide as health foods of the 21st century. *Tricholoma* species produce various types of secondary metabolites, which have been extensively studied by the scientific community. In 2018, Clericuzio et al. summarized the structures, biosynthesis, and biological activities of over one hundred different secondary metabolites isolated from the fruiting bodies of 25 *Tricholoma* species. Building on this, the present article reviews the research progress on *Tricholoma* secondary metabolites from 2018 to 2023, identifying a total of 101 compounds, 46 of which were newly discovered. These secondary metabolites include a wide range of chemical categories such as terpenoids, steroids, and alkaloids, demonstrating broad biological activities. This article aims to provide in-depth scientific insights and guidance for researchers in this field by summarizing the chemical and biological properties of these secondary metabolites, promoting further applications and development of *Tricholoma* fungi in the pharmaceutical and food industries.

## 1. Introduction

In recent years, with the increasing demand for health and nutrition, more and more mushrooms have gained attention in the food and medical fields. *Tricholoma*, an ancient and important edible mushroom, has been used as a medicinal material by humans since ancient times. *Tricholoma* is a fungus classified under the Basidiomycota class, the Tricholomataceae family, and the *Tricholoma* genus. It is particularly notable for its abundance of secondary metabolites, including polysaccharides, alkaloids, and phenolic compounds [1,2]. These secondary metabolites not only confer unique biological activities to *Tricholoma*, but also exhibit broad application potential. For example, *Tricholoma* polysaccharides have been found to possess whitening effects [3], *Tricholoma* peptides show immunomodulatory activity [4], and *Tricholoma* sterols demonstrate good anti-acetylcholinesterase (AchE) activity [5]. Although the secondary metabolites of *Tricholoma* have attracted widespread attention, research is still at a stage that requires further investigation. As the understanding of *Tricholoma* secondary metabolites continues to grow, the potential of these metabolites within an important medicinal and edible fungus will be better explored and utilized.

*Tricholoma* contains a rich array of metabolites, sparking extensive exploration by scientists. In 1968, German scientists first discovered that *Tricholoma* species produce an unpleasant odor. Using thin-layer chromatography, they preliminarily isolated and identified indole as the main component responsible for the bitter smell in *Tricholoma* fruiting bodies [2]. With advances in research techniques and methods, more secondary metabolites from *Tricholoma* have been discovered. In 2018, Italian scientist Clericuzio and colleagues summarized 112 secondary metabolites isolated from *Tricholoma* fruiting bodies (excluding mycelium) [1]. Since then, a large number of secondary metabolites have continued to be discovered in *Tricholoma*. Based on this, the present review summarizes the secondary metabolites of *Tricholoma* identified between 2018 and 2023, that is, a total of 101 compounds discovered, including 46 novel compounds. This article provides a comprehensive summary of the isolation, structural characteristics, and biological activities of these chemical substances. Table 1 lists the secondary metabolites found in *Tricholoma* from 2018 to 2023 and their biological activities.

## 2. Secondary Metabolites of *Tricholoma*

### 2.1. Terpenoids

Terpenoids are the largest and most structurally diverse class of natural products among secondary metabolites [6]. These compounds are based on isoprene units (C5 units) as their fundamental building blocks, resulting in rich structural diversity and widespread distribution. In the past five years, 73 terpenoid compounds have been identified from the genus *Tricholoma*, including triterpenes, diterpenes, and, less commonly, sesquiterpenes and C17 compounds. It is noteworthy that while sesquiterpenes are quite common in other species, they are rarely reported in *Tricholoma*. This suggests that *Tricholoma* may have unique evolutionary strategies in its secondary metabolic pathways.

#### 2.1.1. Triterpenes and Sterols

Triterpenes are a class of secondary metabolites widely found in nature, typically derived from the acyclic precursor squalene [7]. Fungi are significant sources of triterpenes and steroid compounds. These compounds not only possess diverse pharmacological activities but are also crucial for new drug discovery. In the past five years, a total of 64 steroid or triterpene compounds have been identified from *Tricholoma*.

Lanostanes are a class of tetracyclic triterpenes with a core structure consisting of 30 carbon atoms. In this structure, the methyl group at C(13) is in the β-configuration, while the methyl group at C(14) is in the α-configuration, and the C(17) position is connected to a β-configured side chain [8]. Lanosterol is one of the most representative compounds among lanostanes, playing a critical role in triterpene biosynthesis as an important intermediate for the synthesis of sterol compounds such as ergosterol and stigmasterol. Ergosterol is a crucial component of the cell membranes of most fungi and serves as a precursor for vitamin D2 [9], playing an essential role in maintaining fungal life activities. Stigmasterol has cholesterol-lowering, antioxidant, and potential anti-cancer properties [10].

**Figure 1:**
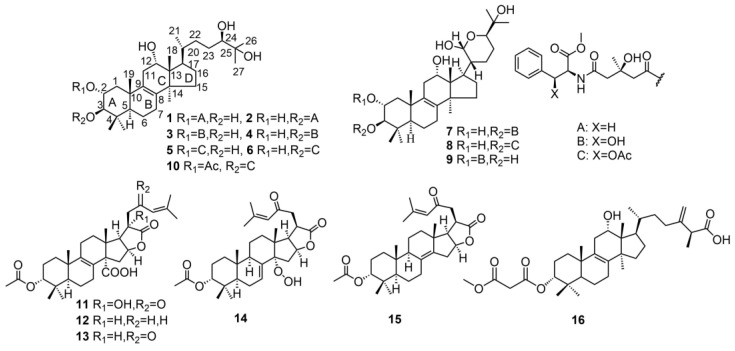
Chemical structures of the lanostane triterpenoids.

Zhang et al. [11] isolated eight novel lanostane-type triterpenes, named pardinols A–H (**1–8**), along with a known compound saponaceol B (**9**) from the fruiting bodies of the fungus *T. pardinum.* These compounds have a characteristic lanostane triterpene skeleton, and the A-ring contains a 3-hydroxy-3-methylglutaryl (HMG) group [12]. This structural feature is uncommon among triterpenes in other species. The authors elucidated the structures of these compounds through extensive spectroscopic analyses, including nuclear magnetic resonance (NMR) and mass spectrometry (MS), and applied alkaline methanolysis to reveal further structural details. The final structural and stereochemical configurations were successfully established using time-dependent density functional theory (TDDFT) and electronic circular dichroism (ECD) calculations, completing comprehensive structural identification [11]. Gozzini et al. [13] isolated three lanostane triterpenes from the ethyl acetate extract of *T. saponaceum* fruiting bodies, including a newly discovered compound, saponaceol D (**10**), and two previously reported compounds from *T. pardinum*, pardinol B (**2**) and pardinol D (**4**). The structures of these compounds were determined through spectrometric analysis [11,13]. Gilardoni et al. isolated three compounds, tricholidic acids B (**11**), C (**12**), and tricholidic acid (**13**) from the ethyl acetate extract of *T. ustaloides* fruiting bodies collected from Italian beech wood. Compounds **11** and **12** were identified for the first time [14]. Zhang et al. isolated three lanostane triterpenes (**14–16**) from the fruiting bodies of *T. imbricatum*, including two novel compounds, tricholimbrins A (**14**) and B (**15**). It is worth noting that **11–15** are all pentacyclic triterpenoids containing unique 5,5-*cis*-fused γ-lactone structures to the D ring [15] (Figure 1).

Ergostane-type compounds are natural products whose biosynthesis originates from lanosterol. This conversion process involves several key steps: first, the removal of three methyl groups from the C(14) and C(4) positions of lanosterol, followed by the introduction of a new methyl group at the C(24) position, forming the basic skeleton of ergostane [8]. These compounds are widely distributed in the fungal. Ergosterol can undergo various reactions such as oxidation, carbon-carbon bond cleavage, ring expansion, or ring contraction to produce new derivatives.

Zhang et al. identified a total of 29 ergostane compounds from the fruiting bodies of *T. imbricatum,* including 26 previously reported compounds (**17–42**) and three newly discovered ergosterol derivatives: tricholimbrins C, D, and E (**43–45**). These new compounds include a ring-rearranged ergosterol derivative, a highly conjugated ergosterol, and a novel ergosterol derivative. The discovery of these compounds provides new insights into the biosynthesis and metabolic pathways of ergosterol [15].

**Figure 2:**
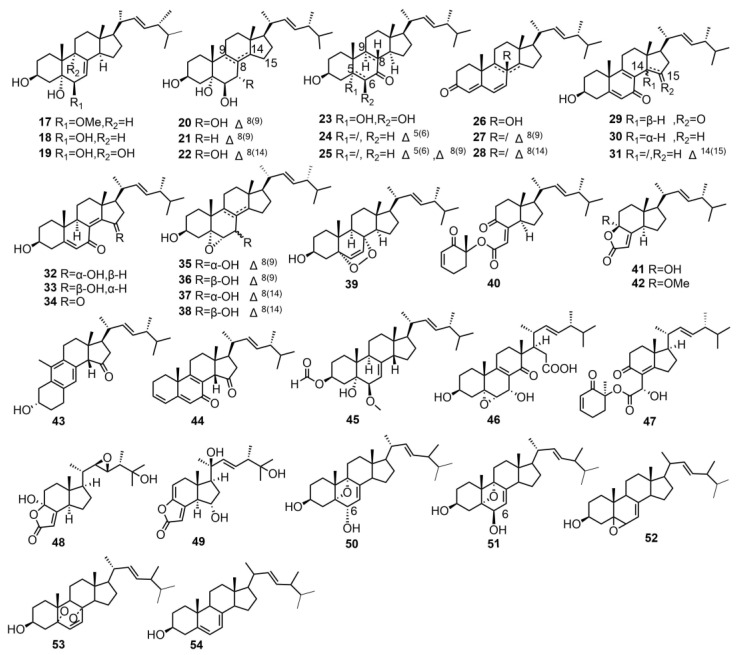
Chemical structures of the ergostane triterpenoids.

Jin et al. isolated four unique ergosterol derivatives from the fruiting bodies of *T. terreum*, named tricholosterols A–D (**46–49**), among these, compound **46** exhibited a rare D-ring open ergosterol skeleton, while **47–49** were identified as rare degraded ergosterols. These characteristics are extremely uncommon among known ergosterol derivatives. This was the first report of such compounds from this species, marking a significant breakthrough in the study of steroid components in *T. terreum* [16]. Kaplaner et al. isolated two novel compounds from the acetone extract of *T. anatolicum*, anatoluin A (**50**) and anatoluin B (**51**). The study confirmed that the -OH substituent at the 6-position has an α-configuration in **50** and a β-configuration in **51**. These compounds belong to a new class of natural ergosterol derivatives. Additionally, three previously reported ergosterols (**52–54**) were also isolated [17].The structures of these compounds are shown in Figure 2.

**Figure 3:**
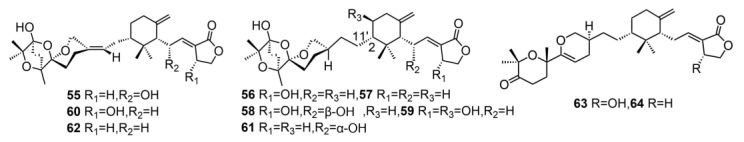
Chemical structures of the triterpenoids.

*Tricholoma* triterpenoids are a unique class of triterpenoid natural products produced by the fungi of the genus *Tricholoma*. These structures include a central methylene cyclohexane ring connected to two heterocycles, along with a γ-lactone substituent and a spiroketal carbon shared by the pyran and bridged heterocyclic system [18,19]. Gozzini et al. [13] isolated a new triterpenoid compound, saponaceolide T (**55**), from the ethyl acetate extract of *T. saponaceum* fruiting bodies, along with the previously reported C-30 terpenoid compounds, saponaceolides A–D (**56–59**), F (**60**), and H (**61**). The structures of these compounds were determined through spectrometric analysis [13]. Compounds **56–59** possess an unprecedented C-30 terpenoid skeleton, which may be assembled through a novel biosynthetic pathway that does not involve the classical cyclization of oxidosqualene to triterpene precursors. Instead, they are composed of two C15 units connected via a C(11′)-C(2) bond, and are more accurately described as sesquiterpene dimers. Gilardoni et al. discovered two previously reported triterpenoid compounds, saponaceolides F (**60**) and J (**62**), in *T. ustaloides* [14]. Chen et al. isolated two novel triterpenoid compounds, tricholopardin C (**63**) and D (**64**), from *T. pardinum* [18]. These compounds feature four separate rings and exhibit a novel linear structure, which might possess potential biological activities. The structures of these compounds are shown in Figure 3.

#### 2.1.2. Diterpenoids

Diterpenoids, as a representative class of terpenoids, are widely present in plants but rarely appear in higher fungi. Among large fungi, the most abundant diterpenoids are cyathane-type diterpenoids, which possess a unique 5–6–7 tricyclic carbon skeleton with a trans 6–7 ring junction [20]. The genus *Cyathus* (*Nidulariaceae*) is a significant producer of cyathane-type diterpenoids [21]. In the past five years, four compounds identified as novel diterpenoids have been discovered in the fruiting bodies of *Tricholoma*. These compounds exhibit a rare and unique rearranged terpenoid skeleton, expanding the diversity of known diterpenoids produced by Basidiomycetes [22].

**Figure 4:**
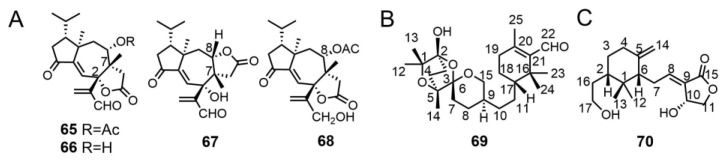
Chemical structures of the diterpenoids (**A**), sesterterpene (**B**), and the C17 compound (**C**).

Gilardoni et al. was the first to isolate and identify four novel diterpenoid compounds, tricholomalides D–G (**65–68**), from the fruiting bodies of *T. ustaloides*. The absolute configurations of these compounds were determined through detailed analysis of MS, NMR, and circular dichroism (CD) spectrometric data. Compounds **65**, **66**, and **68** feature a γ-lactone ring fused in a *cis* manner at C-2 and C-7 to the central cycloheptene ring of the second steroidal skeleton, while tricholomalide F (**67**) has the γ-lactone ring fused in a cis manner at C-7 and C-8 to the cycloheptene ring [23]. This characteristic is extremely rare among natural products (Figure 4A).

#### 2.1.3. Sesterterpenoids and C17 Compounds

Sesterterpenoids, as a rare class of terpenoid natural products, have garnered significant attention due to their unique biological activities, such as anti-inflammatory [24], antibacterial [25], and anti-cancer properties [26]. However, these compounds constitute less than 2% of the total number of terpenoids [27], making them some of the rarest terpenoid compounds to date [28], with only about 1500 compounds formally reported [29]. Most sesterterpenoid compounds have been successfully extracted and isolated from sponges [30]. Notably, only one sesterterpenoid has been discovered from *Tricholoma* and, during this process, a rare C17 compound was also unexpectedly found.

Feng et al. [31] isolated a novel C25 sesterterpenoid compound, tricholopardin A (**69**) from the fruiting bodies of *T. pardinum* (Figure 4B). Additionally, they isolated a C17 compound, tricholopardin B (**70**), which is quite uncommon in fungi (Figure 4C). This suggests that *Tricholoma* may possess unique biosynthetic pathways. The structures of these compounds were determined using spectroscopic methods, electronic circular dichroism, and optical rotatory dispersion calculations [31].

### 2.2. Alkaloids

Alkaloids are a class of nitrogen-containing organic compounds that are widely distributed in nature and possess significant bioactivity [32]. Based on their core chemical structures, alkaloids are classified into several types, including isoquinoline, quinoline, indole, and piperidine alkaloids [33]. The chemical structures of alkaloids are diverse and complex, and they exhibit numerous pharmacological activities, including anti-inflammatory [34], antibacterial [35] and anti-cancer properties [36]. They are widely found in traditional Chinese medicinal herbs, with their medicinal functions gradually being elucidated. In recent years, four alkaloid compounds have been discovered in *Tricholoma*, two of which are newly identified.

#### 2.2.1. Diketopiperazine

Diketopiperazine alkaloids (DKPs) are a unique class of compounds formed by the cyclization of two amino acids into cyclic dipeptides [37]. As an important component of alkaloids, DKPs are relatively common in the fermentation broths of higher fungal species but are quite rare in the fruiting bodies [37,38].

**Figure 5:**
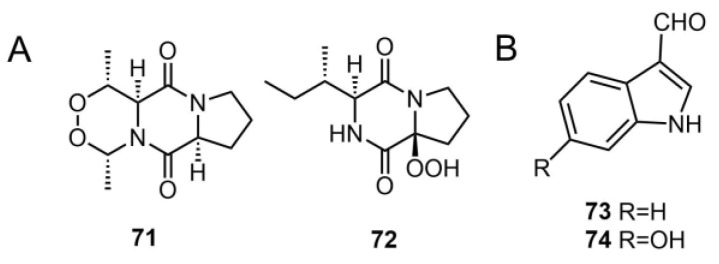
Chemical structures of the diketopiperazine derivatives (**A**) and indole derivatives (**B**).

Zhao et al. isolated two novel diketopiperazine compounds, matsudipeptides A (**71**) and B (**72**) [39], from the fruiting bodies of *T. matsutake*. Both compounds contain a peroxide group (O-O), with **71** featuring a peroxide bridge structure. In this structure, two oxygen atoms are connected by sharing one oxygen atom, forming a bridge that links two carbon atoms, an unusual structure related to the biosynthesis of cyclic dipeptides. Natural products containing endoperoxide are not very common and the structure types containing endoperoxide are always terpenoids, polyketides, or ergosterols [39,40]. Notably, this is the first report of a diketopiperazine with peroxy groups [39]. This study enhanced the chemical diversity of diketopiperazines derived from mushrooms (Figure 5A).

#### 2.2.2. Indole Derivatives

The main components responsible for the bitter odor produced by the fruiting bodies of *Tricholoma* species are indole derivative compounds [1]. Clericuzio et al. isolated two previously reported indole compounds, **73** and **74** [41], from the mushroom *T. pardinum*. Compound **73** was previously found in *T. sulphureum* and *T. matsutake* [1], while **74** was isolated from *Agrocybe cylindracea* [42]. The unpleasant odor emitted by many *Tricholoma* fruiting bodies is likely due to their significant abundance of indole derivatives, which exhibit monomeric and dimeric structural characteristics [43]. Similar odors have been detected in *T. album* and *T. sulphureum* [1,41] (Figure 5B).

### 2.3. Other Compounds

#### 2.3.1. γ-Glutamine Derivative

In 1990, Eizenhofer et al. isolated the compound lascivol (**75**) from the MeOH extract of freeze-dried fruiting bodies of *T. lascivum* [44]. Until 2020, the absolute configuration of the hydroxyl group at the C3 position of **75** was not confirmed and was re-determined by Oba et al. using an improved Mosher method [45]. Lascivol is a new representative of γ-glutamine derivatives commonly found in Basidiomycetes [44].

#### 2.3.2. Amide Derivatives

**Figure 6:**
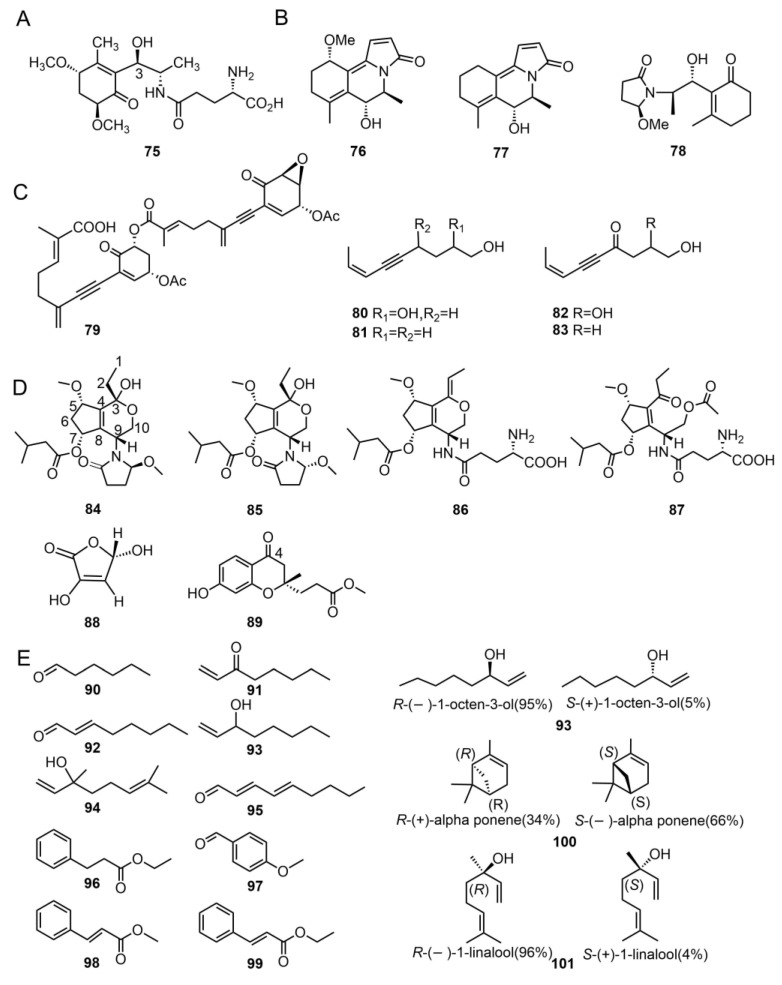
Chemical structures of the γ-glutamine derivative (**A**), amide derivatives (**B**), acetylene compounds (**C**), polyketide compounds (**D**), and volatile compounds (**E**).

Amide derivative compounds play an important role in natural products. For example, β-lactam antibiotics, such as penicillin compounds, inhibit bacterial cell wall synthesis, making them widely used in the medical field to treat bacterial infections [46]. Zhang et al. isolated two new amide compounds, tricholomines A (**76**) and B (**77**), from the dried fruiting bodies of *T. bakamatsutake*. The absolute configuration of these compounds was confirmed by single-crystal X-ray diffraction analysis [47]. In 2023, the same research team isolated another new amide compound, tricholomine C (**78**) [48] (Figure 6B).

#### 2.3.3. Acetylene Compounds

In nature, alkynyl compounds are composed of molecules containing one, two, or more triple bonds, and these naturally occurring compounds are often referred to as acetylene compounds [49]. Their distinctive feature is the presence of an alkynyl group (-C≡C-), which can be part of linear or cyclic structures. Alkynyl compounds have a broad range of sources, and various alkynyl metabolites have been isolated and identified from plants, fungi, and other organisms [50].

Gilardoni et al. isolated a previously reported alkynyl compound, tricholomenyn C (**79**) from *T. ustaloides* [14]. Compound **79** is a fragile compound that easily decomposes in solutions containing trace inorganic acids. It is also the first naturally occurring dimeric enynylcyclohexenone [51]. Clericuzio et al. isolated four unprecedented acetylenic alcohols from *T. pardinum*: (*Z*)-non-7-en-5-yn-1,2,4-triol (**80**), (*Z*)-non-7-en-5-yn-1,4-diol (**81**), (*Z*)-1,2-dihydroxynon-7-en-5-yn-4-one (**82**), and (*Z*)-1-hydroxynon-7-en-5-yn-4-one (**83**) [41]. These compounds feature a nine-carbon linear alkynyl chain, with terminal hydroxyl modifications. The structures, names, and sources of these compounds are shown in Figure 6C and Table 1.

#### 2.3.4. Polyketide Compounds

Yang et al. [52] isolated four polyketide amino acid derivatives, pardinumones A–D (**84–87**) from *T. pardinum*. These compounds feature a combination of a C10 polyketide unit and an amino acid (or its derivative) unit, which is uncommon in natural sources. The structures and absolute configurations of these compounds were determined through spectral data analysis, including electronic circular dichroism (ECD) and nuclear magnetic resonance (NMR) analysis [52].

Kaplaner et al. isolated a previously reported polyketide compound, 3,5-dihydroxyfuran−2(5H) −one (**88**), from *T. anatolicum* [17]. Zhang et al. isolated a new 4−chromanone (**89**) from the fruiting bodies of *T. imbricatum* [15]. Compound **89** was identified as a chromanone derivative containing hydroxyl, methyl, and ester groups, with a structure formed by connecting a benzene ring and a cyclohexane ring to form a cyclopentyl ketone (chromanone) scaffold. The chroman-4-one framework belongs to oxygen-containing heterocyclic structures and is a major component of a large class of medicinal compounds, exhibiting a variety of notable biological and pharmacological activities [53]. Their structures are displayed in Figure 6D.

#### 2.3.5. Volatile Compounds

Murray et al. [54] analyzed the wild mushroom *T. magnivelare*, which has a unique aroma, using solvent-assisted flavor evaporation and aroma extract dilution analysis techniques. They detected 12 active components (**90–101**). The study showed that hexanal (**90**), 1-octen-3-one (**91**) or 1-octen-3-ol (**93**), (2*E*,4*E*)-non-2,4-dienal (**95**), (*E*)-methyl cinnamate (**98**), and linalool (**101**) are crucial for the aroma of *T. magnivelare*, and that the content of other active compounds is low. Chiral chromatography showed that alpha-pinene (**100**) is a mixture of 34% (*R*)-(+)- and 66% (*S*)-(–)-enantiomers, while 1-octen-3-ol (**93**) is a mixture of 95% (*R*)-( −)- and 5% (*S*)-(+)-enantiomers and linalool (**101**) is a mixture of 96% (*R*)-(–)- and 4% (*S*)-(+)-enantiomers. These findings lay the groundwork for further studies on the aroma chemistry of other *Tricholoma* compounds [54]. The structures of these compounds are shown in Figure 6E.

## 3. Biological Activity

**Figure 7:**
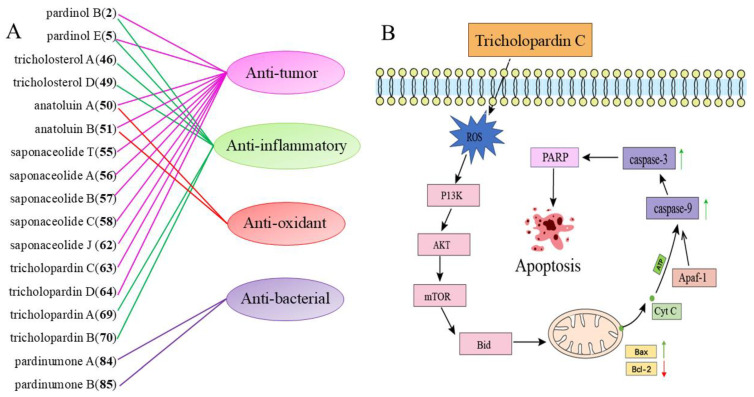
Biological activities of some of the compounds (**A**) from *Tricholoma* and the proposed model of Tricholopardin C induced apoptosis (**B**).

*Tricholoma*, one of the most renowned higher fungi, is rich in bioactive compounds. Extracts from *Tricholoma* contain various active components with antioxidant, anti-inflammatory, and antibacterial properties [1]. Among the numerous secondary metabolites in *Tricholoma*, anatoluin A (**50**) and B (**51**) exhibit multiple bioactive functions. The majority of compounds found in *Tricholoma* have demonstrated anti-cancer properties. The biological activities of the secondary metabolites of *Tricholoma* are shown in Table 1 and Figure 7A.

### 3.1. Antibacterial Activity

The polyketide–amino acid derivatives **84–87** exhibit moderate antibacterial activity against *Staphylococcus aureus*, *Staphylococcus epidermidis*, and *Escherichia coli*, with MIC values ranging from 6.25 to 50 μg/mL [52].

Similarly, the most abundant volatile compound in *Tricholoma*, **93**, can alter cell membrane permeability and exhibits strong antibacterial activity against various bacteria, including *S. aureus*, *Bacillus subtilis*, *S. epidermidis*, and *E. coli* [55,56].

Karakas et al. determined the significant antibacterial potential of the methanol crude extract of *T. terreum* against *S. epidermidis*, *Serratia marcescens*, and *Proteus vulgaris* using the disk agar diffusion method (Kirby-Bauer method) [57].

Additionally, experiments by Li et al. demonstrated that **40** possesses antibacterial activity against *S. aureus*, *B. subtilis*, *Pseudomonas aeruginosa*, *S. epidermidis*, and *E. coli*, but it had a more significant inhibitory effect on *S. aureus* and *B. subtilis* [58].

### 3.2. Anti-Cancer Activity

Compounds **2**, **5–8**, **26**, **32**, **41**, and **42** inhibited the growth of human tumor cell lines HL-60, SMMC-7721, A-549, MCF-7, and SW480, with IC_50_ values all being below 40 μM [11,15]. Compounds **29** and **30** showed selective cytotoxicity against HL-60 and A-549, while **40** and **16** exhibited selective toxicity against A-549 and MCF-7. Notably, **29** and **30** had IC_50_ values below 10 μM against HL-60, while **40** and **42** showed IC_50_ values below 10 μM against A-549, demonstrating significant inhibitory effects. Structure–activity relationship analysis revealed that triterpenoids and ergosteroids with conjugated systems, such as **26**, **32**, and **40–42**, exhibited cytotoxicity, providing new insights for anti-cancer drug development [15].

Compounds **50–53** demonstrated significant cytotoxic activity against four different cancer cell lines (MCF7, HT29, H1299, and HeLa). Based on the cytotoxicity results, compounds **50**, **51**, and **52** selectively inhibited MCF-7 and H1299 cancer cells, while compounds **50**, **52**, and **53** selectively inhibited HT29 cancer cells, exhibiting good cytotoxic activity. Compound **50** showed the strongest toxicity against the HeLa cell line (with the lowest IC_50_ of 14.3 ± 1.7 μg/mL), and relatively weaker toxicity against the H1299 cell line (IC_50_ of 34.3 ± 1.1 μg/mL). Compound **51** also exhibited good cytotoxic activity similar to **50**. Compounds **50–54** displayed almost no toxicity to normal or healthy cell lines (such as PDF and L929) even at high concentrations (e.g., >100 μg/mL or IC_50_ of 93.2 ± 2.5 μg/mL), indicating they do not cause significant harm to these cells [17]. However, **53** exhibited toxicity to PDF and L929 cells, with IC_50_ values of 52.8 ± 0.5 and 65.6 ± 2.2 μg/mL, respectively [17].

Compounds **56**, **57**, **60**, and **62** exhibited IC_50_ values ranging from 0.3 to 1.5 μM against HL-60 cells, demonstrating significant cytotoxic effects on these cells. Additionally, these compounds showed notable inhibitory effects on A-549, Hep G2, Caki-1, and MCF-7 cells, further confirming their potential in cancer treatment [14]. Compounds **55**, **56**, **58**, **60**, and **61** displayed strong antiproliferative effects on A549, Hep G2, Caki-1, MCF-7, and WISH cells, particularly surpassing the inhibitory effects of the well-known cytotoxic agent shikonin on A549 and Hep G2 cells. Among these, **56**, **60**, and **61** exhibited the most significant cytotoxicity, highlighting their potential application in the development of anticancer drugs [13].

Tricholopardin C (**63**) and **64** exhibited cytotoxicity against MCF-7 and HeLa cell lines. Notably, **63** shows a potent inhibitory effect on MCF-7 cells with an IC_50_ value of 4.7 μM [18]. Research indicates that caspases play a crucial role in tricholopardin C-induced apoptosis. Compared to the control group, activation of caspase-3 and caspase-9 was observed. The activated caspase-3 subsequently promotes the cleavage of PARP protein, leading to apoptosis. Figure 7B reveals the mechanism by which tricholopardin C induces tumor cell death through apoptosis [18].

### 3.3. Anti-Inflammatory Activity

The anti-inflammatory activity of several compounds was evaluated by inhibiting nitric oxide (NO) production in lipopolysaccharide (LPS)-induced RAW264.7 macrophages. Compounds **46** and **49** exhibited moderate inhibitory activity against NO production with IC_50_ values of 27.6 and 31.8 μM, respectively [16]. Compounds **2** and **5–8** exhibited stronger inhibitory effects, with IC_50_ values ranging from 5.3 to 14.70 μM [11]. Compound **69** showed potent NO inhibition with an IC_50_ value of 0.08 μM, indicating significant anti-inflammatory activity, while compound **70** displayed moderate anti-inflammatory activity with an IC_50_ value of 16.2 μM [31].

### 3.4. Antioxidant Activity

The antioxidant activity was determined using lipid peroxidation inhibitors and ABTS^•+^ scavenging activity. Compounds **50–54** exhibited very close activities in both acetone and methanol extracts. Among them, **50** was the most active compound in lipid peroxidation inhibitory activity, followed by **51**, **52**, **53**, and **54**, respectively. In the ABTS assay, the IC_50_ values of **50–54** were all more than 100 μg/mL, indicating relatively weak antioxidant activity [17]. Meanwhile, fungal studies on antioxidant activity suggest that *T. ustale* has a high free radical scavenging effect [59].

**Table 1:** The secondary metabolites of *Tricholoma*, discovered during 2018–2023.

Numbers	Names	Species	Bioactivities	References
Lanostane triterpenoids				
**1**	pardinol A	*T. pardinum*	-	[11]
**2**	pardinol B	*T. pardinum*	Anti-inflammatory activity; Anti-cancer activity	[11]
**3**	pardinol C	*T. pardinum*	-	[11]
**4**	pardinol D	*T. pardinum*	-	[11]
**5**	pardinol E	*T. pardinum*	Anti-inflammatory activity; Anti-cancer activity	[11]
**6**	pardinol F	*T. pardinum*	Anti-inflammatory activity; Anti-cancer activity	[11]
**7**	pardinol G	*T. pardinum*	Anti-inflammatory activity; Anti-cancer activity	[11]
**8**	pardinol H	*T. pardinum*	Anti-inflammatory activity; Anti-cancer activity	[11]
**9**	saponaceol B	*T. pardinum*	-	[11]
**10**	saponaceol D	*T. saponaceum*	-	[11]
**11**	tricholidic acid B	*T. ustaloides*	-	[14]
**12**	tricholidic acid C	*T. ustaloides*	-	[14]
**13**	tricholidic acid	*T. ustaloides*	-	[14]
**14**	tricholimbrin A	*T. imbricatum*	-	[15]
**15**	Tricholimbrin B	*T. imbricatum*	-	[15]
**16**	(25*S*)-(+)-12α-hydroxy-3α-methylcarboxyacetate-24-methyllanosta-8,24(31)-diene-26-oic acid	*T. imbricatum*	Anti-cancer activity	[15]
Ergostane triterpenoids				
**17**	3β,5α-dihydroxy-6β-methoxyergosta-7,22-diene	*T. imbricatum*	-	[15]
**18**	(22*E*,24*R*)-5α,6α-epoxyergosta-8,22-	*T. imbricatum*	-	[15]
**19**	dien-3β,7α-diol	*T. imbricatum*	-	[15]
**20**	(22*E*,24*R*)-ergosta-7,22-diene-3β,5α,6β,9α-tetraol	*T. imbricatum*	-	[15]
**21**	(22*E*,24*R*)-ergosta-8,22-diene-3β,5α,6β,7α-tetrol	*T. imbricatum*	-	[15]
	(22*E*,24*R*)-ergosta-8,22-diene-3β,5α,6β,7α-tetrol	*T. imbricatum*	-	[15]
**22**	(22*E*,24*R*)-ergosta-8(14),22-diene-3β,5α,6β,7α-tetrol	*T. imbricatum*	-	[15]
**23**	3β,5α,6β-trihydroxy-(22*E*,24*R*)-ergost-22-en-7- one	*T. imbricatum*	-	[15]
**24**	3β-hydroxy-(22*E*,24*R*)-ergosta-5,22- dien-7-one	*T. imbricatum*	-	[15]
**25**	3β-hydroxy-(22*E*,24*R*)-ergosta-5,22- dien-7-one	*T. imbricatum*	-	[15]
**26**	isocyathisterol	*T. imbricatum*	Anti-cancer activity	[15]
**27**	(22*E*)-ergosta-4,6,8,22-tetraen-3-one	*T. imbricatum*	-	[15]
**28**	(22*E*,24*R*)-ergosta-4,6,8(14),22-tetraen-3-one	*T. imbricatum*	-	[15]
**29**	3β-hydroxyl-(22*E*,24*R*)-ergosta-5,8,22-trien-7,15-dione	*T. imbricatum*	Anti-cancer activity	[15]
**30**	3β-hydroxyl-(22*E*,24*R*)-ergosta-5,8,22-trien-7-one	*T. imbricatum*	Anti-cancer activity	[15]
**31**	3β-hydroxyl-(22*E*,24*R*)- ergosta-5,8,14,22-tetraen-7-one	*T. imbricatum*	-	[15]
**32**	3β,15α-dihydroxyl-(22*E*,24*R*)-ergosta-5,8(14),22-trien-7-one	*T. imbricatum*	Anti-cancer activity	[15]
**33**	3β,15β-dihydroxyl-(22*E*,24*R*)-ergosta-5,8(14),22-trien-7-one	*T. imbricatum*	-	[15]
**34**	3β-hydroxyl-(22*E*,24*R*)-ergosta-5,8(14),22-trien-7,15-dione	*T. imbricatum*	Anti-cancer activity	[15]
**35**	5α,6α-epoxy-(22*E*,24*R*)-ergosta-8,22-diene-3β,7β-diol	*T. imbricatum*	Anti-cancer activity	[15]
**36**	5α,6α-epoxy-(22*E*,24*R*)-ergosta-8(14),22-diene-3β,7α-diol	*T. imbricatum*	-	[15]
**37**	5α,6α-epoxy-(22*E*,24*R*)-ergosta-8(14),22-diene-3β,7β-diol	*T. imbricatum*	-	[15]
**38**	5α,6α-epoxy-(22*E*,24*R*)-ergosta-8(14),22-diene-3β,7β-diol	*T. imbricatum*	-	[15]
**39**	5α,8α-epidioxy-(22*E*,24*R*)-ergosta-6,22- dien-3β-ol	*T. imbricatum*	-	[15]
**40**	chaxine C	*T. imbricatum*	Antibacterial activity; Anti-cancer activity	[15,58]
**41**	demethylincisterol A3	*T. imbricatum*	Anti-cancer activity	[15]
**42**	volemolide	*T. imbricatum*	Anti-cancer activity	[15]
**43**	tricholimbrin C	*T. imbricatum*	-	[15]
**44**	tricholimbrin D	*T. imbricatum*	-	[15]
**45**	tricholimbrin E	*T. imbricatum*	-	[15]
**46**	tricholosterol A	*T. terreum*	Anti-inflammatory activity	[16]
**47**	tricholosterol B	*T. terreum*	-	[16]
**48**	tricholosterol C	*T. terreum*	-	[16]
**49**	tricholosterol D	*T. terreum*	Anti-inflammatory activity; Cytotoxic against human cancer cell lines	[16]
**50**	anatoluin A	*T. anatolicum*	Antioxidant activity; Cytotoxic against human cancer cell lines	[17]
**51**	anatoluin B	*T. anatolicum*	Antioxidant activity; Cytotoxic against human cancer cell lines	[17]
**52**	5α,6α-epoxy-ergosta-7,22-dien,3β-ol	*T. anatolicum*	Antioxidant activity; Cytotoxic against human cancer cell lines	[17]
**53**	ergosterol-endoperoxide	*T. anatolicum*	Antioxidant activity; Cytotoxic against human cancer cell lines	[17]
**54**	ergosterol,3β-ol	*T. anatolicum*	-	[17]
Triterpenoids				
**55**	saponaceolide T	*T. saponaceum*	Cytotoxic against human cancer cell lines	[13]
**56**	saponaceolide A	*T. saponaceum*	Cytotoxic against human cancer cell lines	[13]
**57**	saponaceolide B	*T. saponaceum*	Cytotoxic against human cancer cell lines	[13]
**58**	saponaceolide C	*T. saponaceum*	Cytotoxic against human cancer cell lines	[13]
**59**	saponaceolide D	*T. saponaceum*	-	[13]
**60**	saponaceolide F	*T. saponaceum*	Cytotoxic against human cancer cell lines	[13,14]
**61**	saponaceolide H	*T. saponaceum*	Cytotoxic against human cancer cell lines	[13]
**62**	saponaceolide J	*T. ustaloides*	Cytotoxic against human cancer cell lines	[14]
**63**	tricholopardin C	*T. pardinum*	Cytotoxic against human cancer cell lines	[18]
**64**	tricholopardin D	*T. pardinum*	Cytotoxic against human cancer cell lines	[18]
Diterpenoids				
**65**	tricholomalide D	*T. ustaloides*	-	[23]
**66**	tricholomalide E	*T. ustaloides*	-	[23]
**67**	tricholomalide F	*T. ustaloides*	-	[23]
**68**	tricholomalide G	*T. ustaloides*	-	[23]
Sesterterpenoid				
**69**	tricholopardin A	*T. pardinum*	Anti-inflammatory activity	[31]
C17 compound				
**70**	tricholopardin B	*T. pardinum*	Anti-inflammatory activity	[31]
Diketopiperazines				
**71**	matsudipeptide A	*T. matsutake*	-	[39]
**72**	matsudipeptide B	*T. matsutake*	-	[39]
Indole derivatives				
**73**	1H-indole-3-carbaldehyde	*T. lascivum*	-	[45]
**74**	6-hydroxy-1H-indole-3-carbaldehyde	*T. pardinum*	-	[41]
γ-glutamine derivative				
**75**	lascivol	*T. pardinum*	-	[41]
Amide derivatives				
**76**	tricholomine A	*T. bakamatsutake*	-	[47]
**77**	tricholomine B	*T. bakamatsutake*	-	[47]
**78**	tricholomine C	*T. bakamatsutake*	-	[48]
Acetylene compounds				
**79**	tricholomenyn C	*T. ustaloides*	-	[14]
**80**	(*Z*)-non-7-en-5-yn-1,2,4-triol	*T. pardinum*	-	[41]
**81**	(*Z*)-non-7-en-5-yn-1,4-diol	*T. pardinum*	-	[41]
**82**	(*Z*)-1,2-dihydroxynon-7-en-5-yn-4-one	*T. pardinum*	-	[41]
**83**	(*Z*)-1-hydroxynon-7-en-5-yn-4-one	*T. pardinum*	-	[41]
Polyketide compounds				
**84**	pardinumone A	*T. pardinum*	Antibacterial activity	[52]
**85**	pardinumone B	*T. pardinum*	Antibacterial activity	[52]
**86**	pardinumone C	*T. pardinum*	Antibacterial activity	[52]
**87**	pardinumone D	*T. pardinum*	Antibacterial activity	[52]
**88**	3,5-dihydroxyfuran-2(5H)-one	*T. anatolicum*	-	[17]
**89**	4-chromone derivative	*T. imbricatum*	-	[15]
Volatile compounds				
**90**	hexanal	*T. magnivelare*	-	[54]
**91**	1-octen-3-one	*T. magnivelare*	-	[54]
**92**	(*E*)-oct-2-enal	*T. magnivelare*	-	[54]
**93**	1-octen-3-ol	*T. magnivelare*	Antibacterial activity	[55,56]
**94**	linalool	*T. magnivelare*	-	[54]
**95**	(2*E*,4*E*)-nona- 2,4-dienal	*T. magnivelare*	-	[54]
**96**	ethyl 3-phenylpropanoate	*T. magnivelare*	-	[54]
**97**	4-methoxybenzaldehyde	*T. magnivelare*	-	[54]
**98**	methyl (E)-3-phenylprop-2-enoate	*T. magnivelare*	-	[54]
**99**	3,4-dimethoxybenzaldehyde	*T. magnivelare*	-	[54]
**100**	α-pinene	*T. magnivelare*	-	[54]
**101**	linalool	*T. magnivelare*	-	[54]

## 4. Conclusions and Prospect

In recent years, with the increasing demand for health and nutrition, mushrooms have garnered significant attention as valuable resources for both medicinal and culinary purposes. Mushrooms are not only popular on dining tables for their taste and nutritional value but also widely used in the medical field due to their various medicinal properties. For instance, *Ganoderma lucidum* is extensively used in health supplements and traditional Chinese medicine because of its notable immune-modulating and anti-cancer effects [60]. *Hericium erinaceus* is renowned for its protective gastric and neuro-regulatory functions, and is commonly used in both traditional medicine and dietary supplements [61,62]. *Lentinula edodes* and *Agrocybe* are known for their rich polysaccharides and alkaloids, which exhibit anti-cancer, antiviral, and immune-regulating effects [42,63]. Similarly, *Tricholoma* has also attracted widespread attention.

This review summarizes the rich research achievements in the field of secondary metabolites of *Tricholoma* from 2018 to 2023. Up to 101 compounds have been isolated and identified, including 46 novel compounds. Most of these compounds are extracted from the fruiting bodies of *Tricholoma* mushrooms, exhibiting diverse structures that highlight the complex metabolic pathways that have evolved in these fungi as products of natural evolution. Of particular interest are compounds such as triterpenes, diterpenes, and rare C17 compounds, which possess unprecedented chemical scaffolds and unique biosynthetic pathways. These findings not only expand our understanding of fungal secondary metabolism mechanisms but also offer possibilities for enhancing the production and diversity of these secondary metabolites through rational modification of host organisms or de novo synthesis methods.

From a biological activity perspective, the secondary metabolites from *Tricholoma* have properties including anti-cancer, anti-inflammatory, anti-microbial, and antioxidant, and demonstrate potential applications in pharmaceutical research. Additionally, crude acetone extracts of *T. pardinum* mushrooms exhibit strong acaricidal activity against dangerous crop pests from the Acarinae family [41], offering new solutions for agricultural biocontrol. In today’s advancing technology landscape, our understanding of mushrooms and natural products continues to deepen, expanding their potential applications in drug development and beyond. The diversity and bioactivity of the *Tricholoma* secondary metabolites represent a significant resource for pharmaceutical research and agricultural biocontrol, providing a foundation for the development of new drugs and effective biocontrol strategies.
